# Influence of short-term dexamethasone-induced stress on behavioral, physiological and reproductive performance of growing rabbit bucks

**DOI:** 10.1186/s12917-025-05140-w

**Published:** 2026-01-03

**Authors:** Fakhri E. El-Azzazi, Shaaban Saad Elnesr, Ibrahim M. M. Ibrahim, Ahmed A. Ali

**Affiliations:** 1 Department of Animal Production and Fish Resources, Faculty of Agriculture, Suez Canal University, Ismailia, 41522 Egypt; 2 Department of Poultry Production, Faculty of Agriculture, Fayoum University, Fayoum, 63514 Egypt; 3 Department of Animal and Poultry Production, Faculty of Agriculture, Arish University, Arish, Egypt; 4 Department of Animal, Poultry and Fish Behaviour and Management, Faculty of Veterinary Medicine, Suez Canal University, Ismailia, 41522 Egypt

**Keywords:** Dexamethasone, Glucocorticoids, Hormones, Semen quality, Induced stress, Rabbits

## Abstract

The purpose of this study was to investigate how growing rabbit bucks responded to stress induced by dexamethasone (DEX). Sixteen New Zealand White male rabbits (4–5 months old, 2.75 ± 0.07 kg) were randomly assigned to four groups. After a one-week preliminary period under the same management, three groups received intramuscular DEX injections for seven consecutive days at doses of 1, 2, and 3 mg/kg body weight (D01, D02, and D03, respectively), while the control group (D00) received saline. Reproductive, behavioral, and physiological stress indicators were assessed throughout the preliminary, treatment, and two- weeks recovery periods. DEX administration induced dose-dependent adverse effects. Significant reductions in body weight gain, testosterone levels, sexual behavior, and semen quality were observed. High doses of DEX produced a great increase in anxiety-related responses in the open field test, and also in the novel object recognition test, they produced a great decrease in chin marking frequencies during the third 5 min habituation, discrimination ratio (RI), and primary object recognition response (PORR); and altered discrimination ratio for both RI and PORR. Physiological stress markers, including triiodothyronine levels, rectal temperature, and pulse rate, increased significantly, particularly in higher dose groups. The neutrophil-to-lymphocyte ratio also rose in a dose-dependent manner, with only partial recovery post-treatment. In conclusion, high-dose DEX administration in growing rabbit bucks negatively affects semen quality, behavior, growth, and hormonal balance, even over short durations. Therefore, glucocorticoids must be used carefully when breeding animals, particularly during sensitive developmental stages.

## Introduction

Stress refers to a variety of abnormal reactions resulting from the internal and external environmental factors in the organism, including ambient temperature, transportation, feeding techniques, nutrient concentration, pollution, drugs and immunity. Stress disrupts the metabolic levels of animals, leading to metabolic disorders or metabolic diseases [1–4]. In farming systems, the excessive pursuit of productivity has increasingly influenced rabbit sensitivity to a variety of stressors. Stress-induced immune suppression happens through two major routes: the hypothalamic-pituitary-adrenal axis (HPA) and autonomic nervous system [5, 6].

Dexamethasone (DEX) is an artificial glucocorticoid analogue with a strong anti-inflammatory effect when used topically [7] or systemically for a short period and at low doses [8]. On the other hand, they have cell-mediated immunosuppressive effects [9, 10] that have been utilized to simulate stress conditions in animal models [11], and poultry [12] by the HPA axis mechanism and change metabolism in animals [13, 14]. Besides, dietary DEX can produce homologous effects like increased corticosterone levels, activating stress-related signaling pathways [15].

In rabbits, the therapeutic dose of DEX is 1–2 mg/kg [16], but under experimental conditions, doses up to 10 mg/kg were administered [17]. The administration of DEX led to altered immune responses through an altered differential white blood cells (WBCs) count indicating a more pronounced stress response through a higher heterophil/lymphocyte (H/L) ratio in the DEX-fed rabbits [18], based on the fact that rabbits are steroid-sensitive for their relative ease of producing lymphoid depletion after the systemic glucocorticoid application [19, 20]. Higher doses of DEX than those routinely used in clinical medicine can lead to long-lasting immune suppression, with adequate immunosuppressive effects achieved by administering 2 mg/kg DEX phosphate three times daily at 6-hour intervals [20]. On the other hand, Mayer et al. [8] reported that DEX-treated rabbits did not show any outward clinical signs of immune suppression denoted by either gastrointestinal signs or respiratory tract infections.

Behavioral tests were used to analyze stress and anxiety in animals, such as the open field test (OFT) [21] and spatial memory tests like the object recognition test [22]. Animals that are not healthy tend to move less within the area of OFT. Also, stressed animals show less activity in the open field and increased stereotypical behavior [23]. Higher stress levels can effectively provoke bouts of purposeless stereotypical grooming [24]. The decrease in behavioral activities was displayed in response to a variety of environmental stresses considered powerful predictors of anxiety and stress responses to a novel context [25]. In contrast, animals were monitored daily for behavioral changes, of which none were noted for either the DEX-treated or control groups, although rabbits maintained a positive weight gain with a total weight increase of 0.5 ± 0.1 kg per treatment group [8].

Dexamethasone is a synthetic glucocorticoid that has seen increasing clinical use in recent years [26, 27]. It is widely prescribed for conditions such as chronic asthma, rheumatoid arthritis, and autoimmune diseases and to prevent graft rejection due to its potent anti-inflammatory, immunosuppressive, and analgesic properties [28, 29]. However, accumulating evidence indicates that DEX has adverse effects on male reproductive function. Dexamethasone reduces luteinizing hormone (LH) secretion, leading to a decline in circulating testosterone levels [30], which in turn may impair epididymal function and sperm maturation [31]. Moreover, Leydig cells express glucocorticoid receptors, suggesting that DEX may exert direct inhibitory effects on testicular steroidogenesis [32]. DEX is also known to increase intracellular reactive oxygen species production in various tissues such as the liver, kidney, vascular endothelial cells, and neural stem cells, resulting in lipid peroxidation and oxidative stress [33, 34]. These oxidative insults have been associated with reduced daily sperm production [35], impaired sperm motility, and inhibited testosterone synthesis through disruption of the hypothalamic–pituitary–gonadal (HPG) axis and direct testicular toxicity [26, 36]. Furthermore, DEX induces the Fas/FasL apoptotic signaling pathway in Leydig and germ cells, contributing to testicular cell apoptosis [37]. DEX treatment has been shown to cause testicular injury, evidenced by significant reductions in spermatogenic cell counts, spermatogenesis indices, daily sperm production, sperm motility, and testosterone levels, as well as increased lipid peroxidation markers such as malondialdehyde [38].

The aim of this research was to investigate the effect of stress induced by short-term dexamethasone injection at varying doses on growing rabbit bucks’ behavior, physiology, and reproductive performance.

## Materials and methods

### Source of the animals

The experiment was carried out at the rabbitry farm and the laboratories of Animal Production Department, Faculty of Agriculture, Suez Canal University, Ismailia Governorate, during the spring season. The experimental animals (New Zealand White rabbits) were procured from the faculty’s dedicated research farm, where they are bred and maintained basically for scientific investigations in accordance with the faculty’s research plan.

### Ethics and consent to participate

The study received approval (SCU-Agr-REC 26/2025) from the Ethical Committee for Animal Experimentation at the Faculty of Agriculture, Suez Canal University, Ismailia, Egypt.

### Animals and management

Sixteen male New Zealand White (NZW) rabbits were used in the present study. The age of rabbits ranged between 4 and 5 months (an average body weight of 2.75 ± 0.07 kg). Animals were individually housed in galvanized wire cages (50 × 50 × 40 cm) and kept continuously under the same managerial and environmental conditions. Rabbits were fed *ad libitum* a basal pelleted ration containing 17% crude protein, 2.8% fat, 12.8% crude fiber, and 2600 kcal digestible energy/kg diet [39] and water was *ad libitum*. The lighting system was 12 hours’ light/12 hours’ dark in the rabbitry.

### Experimental design

The experiment spanned four weeks. Animals were randomly divided into four experimental groups (four males each) for four periods: preliminary, treatment, one-week recovery and two-week recovery (one week each). During the preliminary period, all males were reared with the same managerial conditions and without any treatment. During the treatment period, dexamethasone was intramuscularly injected for 7 successive days with 1, 2 and 3 mg/kg for three treatment groups (D01, D02 and D03, respectively), while the control (D00) group was injected with saline.

### Sexual libido

The time of sexual libido (reaction time) was determined in seconds using a stopwatch at the time of semen collection process. It was the time interval from the initial stimulus as a response to exposure to a female rabbit to the completion of a specific behavioral sequences including sniffing, grooming, mounting and subsequent ejaculation [40–42].

### Semen collection and evaluation

Using artificial vagina of rabbits, semen samples were collected from all males three times weekly during all experimental periods. Percentages of sperm motility were measured using a phase contrast microscope (OPTIKA, XDS-2, Italy). Sperm viability was determined using eosin-nigrosine viability kits according to Boiti et al. [43]. A total of 200 sperm cells were counted under X400 using an inverted (phase contrast) microscope (OPTIKA, XDS-2, Italy). Sperm cell concentration was determined by a Neubauer haemocytometer. Total motile sperm per ml and per ejaculate, sperm output, were calculated from the initial motility, the semen volume, and the sperm concentration data obtained.

### Physiological parameters

Rabbit’s thermoregulatory indices include rectal temperature (RT) and heart rate (HR), which were measured twice a week and taken between 16.00 and 18.00 h of the day. Rectal temperature was recorded directly using a calibrated digital thermometer. The heart rate was determined by placing the hand on the lower left side of the rabbit’s chest to sense the heartbeat. It was counted for 15 s then multiplied by four to get the heart rate of rabbit per min.

### Complete blood count, testosterone and triiodothyronine levels

Blood samples were collected from rabbit’s auricular marginal veins in heparinized tubes between 8:00 and 10:00 am at the end of the preliminary, treatment, and recovery periods. Whole blood samples were sent to the external laboratory for complete blood count (CBC) determination. Plasma was obtained by blood centrifugation for 20 min at 3000 rpm and stored at −20 °C until analysis. According to manufacturer companies, plasma testosterone and triiodothyronine (T3) were analyzed by ELISA kits (DiaMetra, Spello-Perugia, Italy and Assay pro LLC 3400 Harry S Truman Blvd St. Charles, USA, respectively).

### Behavioral tests

#### Open field test

According to Prut and Belzung [21], the open field test is used to examine stereotyped behaviors, anxiety, and locomotion. At the end of the trials’ preliminary and treatment phases, it was carried out on four animals from each rabbit group. In a separate room next to the rabbit cages, the test was carried out in a 2.5 × 2 m area with timber walls that were one meter high and a plastic floor that was separated into nine numbered squares. Each test lasted 10 min in total for each animal. There was a hidding wooden box with a sliding door that was attached to the area for each rabbit. For the first two minutes rabbits were checked for the number of tries (n) and the delay time (sec) it took to enter the arena after opening the sliding door. If rabbit resfused after these 2 min, it was carefully moved into the arena, then shutting the sliding door, and a video network camera (Panasonic^®^ WV-NS202AE) was used to record its behavior for the rest eight minutes. According to Trocino et al. [44] and Ferrante et al. [45], the behaviors that were measured were total displacements, center displacements, exploration, locomotion, running, escape attempts, hopping, standing still, rearing, grooming, digging, biting, resting, defecation, and urination. Between tests, 30% ethanol was used to clean the open field arena. All rabbits participated in a 10-minute recording session using continuous recording and focal sampling techniques. Analysis of every testing session was done using ANY MAZE ^®^ software.

#### Novel object recognition (NOR) test

All novel object recognition experiments were carried out within the open field arena described above and it has three phases. Two objects (plastic cups) were differed in shape and color; therefore, they were easily distinguishable by visual and/or tactile characteristics, as we prevented olfactory cues by always using clean, unmarked cups. In the first (sample phase), rabbits were placed in an arena with two similar objects for 15 min. Then in the second (delay phase) they were returned to their cage for a 5-minute pause. At last, in the third test phase, they were returned to the same arena with one familiar object that was like the original ones and one novel object that was different from the original ones for 15 min. Chin-marks were recorded at each of the objects (familiar and novel) [22].

During the test phases, according to Hoffman and Basurto [22], we measured the Recognition interval (RI) which is the number of chin marks directed at each object (Familiar and Novel) across 5 min. Also, we measure the primary object recognition response (PORR) which is the latency to perform the first chin mark directed at each object and the number of chin marks associated with the rabbit’s first encounter with each object. We use the latter data to calculate the “Discrimination Ratio” for RI and PORR as follows:

$$\begin{aligned}&\:\mathrm{D}\mathrm{i}\mathrm{s}\mathrm{c}\mathrm{r}\mathrm{i}\mathrm{m}\mathrm{i}\mathrm{n}\mathrm{a}\mathrm{t}\mathrm{i}\mathrm{o}\mathrm{n}\:\:\mathrm{r}\mathrm{a}\mathrm{t}\mathrm{i}\mathrm{o}\\&=\frac{\mathrm{N}\mathrm{o}.\:\mathrm{c}\mathrm{h}\mathrm{i}\mathrm{n}\:\:\mathrm{m}\mathrm{a}\mathrm{r}\mathrm{k}\mathrm{s}\:\:\mathrm{o}\mathrm{n}\:\:\mathrm{N}\mathrm{o}\mathrm{v}\mathrm{e}\mathrm{l}-\mathrm{N}\mathrm{o}.\:\:\mathrm{c}\mathrm{h}\mathrm{i}\mathrm{n}\:\:\mathrm{m}\mathrm{a}\mathrm{r}\mathrm{k}\mathrm{s}\:\:\mathrm{o}\mathrm{n}\:\mathrm{F}\mathrm{a}\mathrm{m}\mathrm{i}\mathrm{l}\mathrm{i}\mathrm{a}\mathrm{r}}{\mathrm{T}\mathrm{o}\mathrm{t}\mathrm{a}\mathrm{l}\:\mathrm{n}\mathrm{u}\mathrm{m}\mathrm{b}\mathrm{e}\mathrm{r}\:\:\mathrm{o}\mathrm{f}\:\:\mathrm{c}\mathrm{h}\mathrm{i}\mathrm{n}\:\:\mathrm{m}\mathrm{a}\mathrm{r}\mathrm{k}\mathrm{s}\:\:\mathrm{o}\mathrm{n}\:\:\mathrm{b}\mathrm{o}\mathrm{t}\mathrm{h}\:\:\mathrm{o}\mathrm{b}\mathrm{j}\mathrm{e}\mathrm{c}\mathrm{t}\mathrm{s}}\end{aligned}$$

### Body weight changes

Individual live body weight was recorded weekly and health status was monitored daily to detect any clinical signs of diseases.

### Statistical analysis

Data were analyzed using SPSS Statistics version 27 [46]. To evaluate the effects of different treatments of dexamethasone doses (0, 1, 2, and 3 mg/kg body weight) within each experimental period (pre-treatment, treatment and one-week and two-week recovery), a General Linear Model (GLM) was applied for each period. Multiple comparisons between group means were performed using Duncan’s Multiple Range Test, which is integrated within SPSS. Differences were considered statistically significant at *P* ≤ 0.05.

## Results

### Effect of DEX on sexual libido and testosterone level

**Fig. 1:**
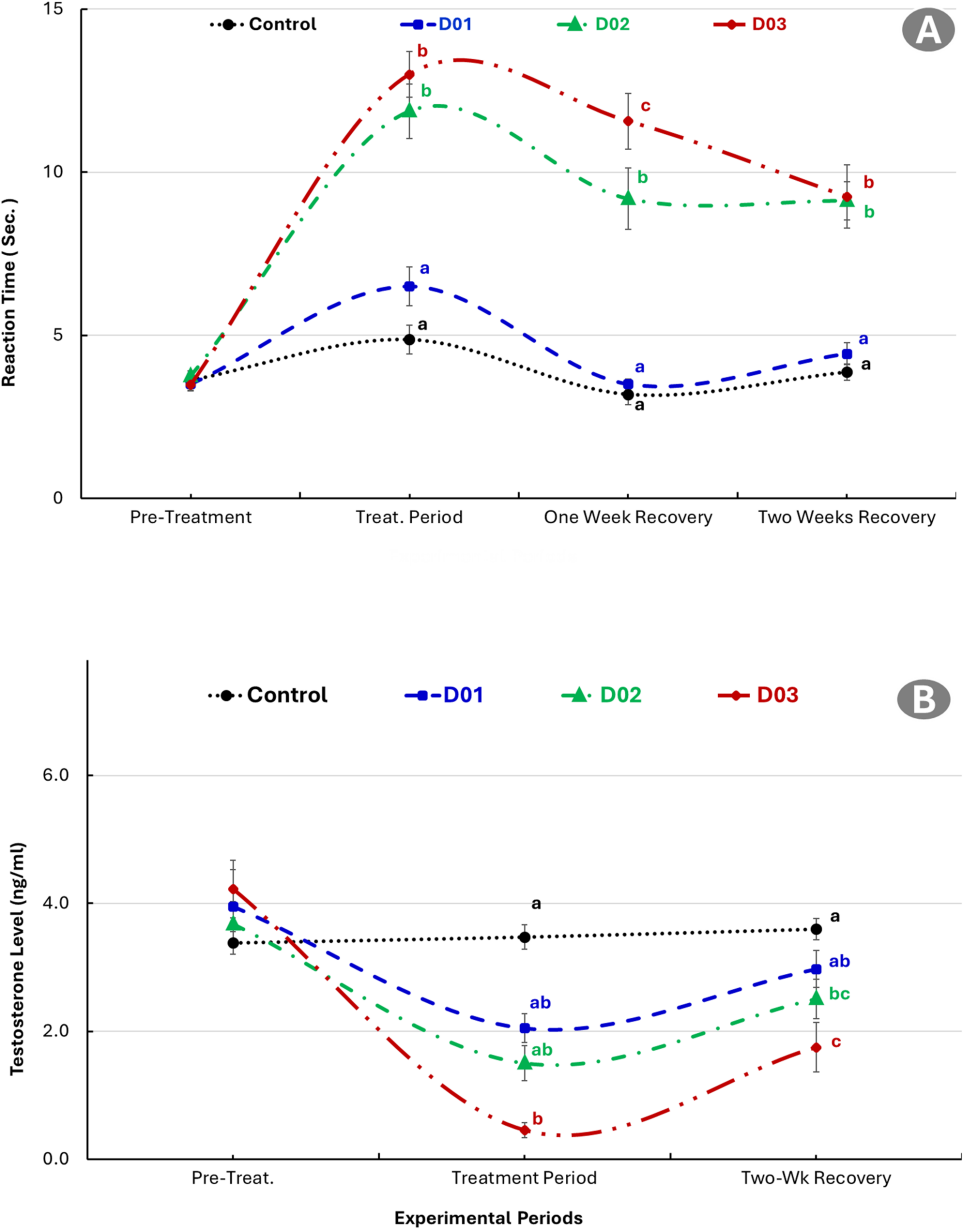
Effect of different doses of dexamethasone (1, 2 and 3 mg/kg) on reaction time (**A**) as an indicator of sexual libido and plasma testosterone level (**B**) in New Zealand White male rabbits. Different letters (a, b, c) denote statistically significant differences (P ≤ 0.05) between groups at the same time point

**Fig. 2:**
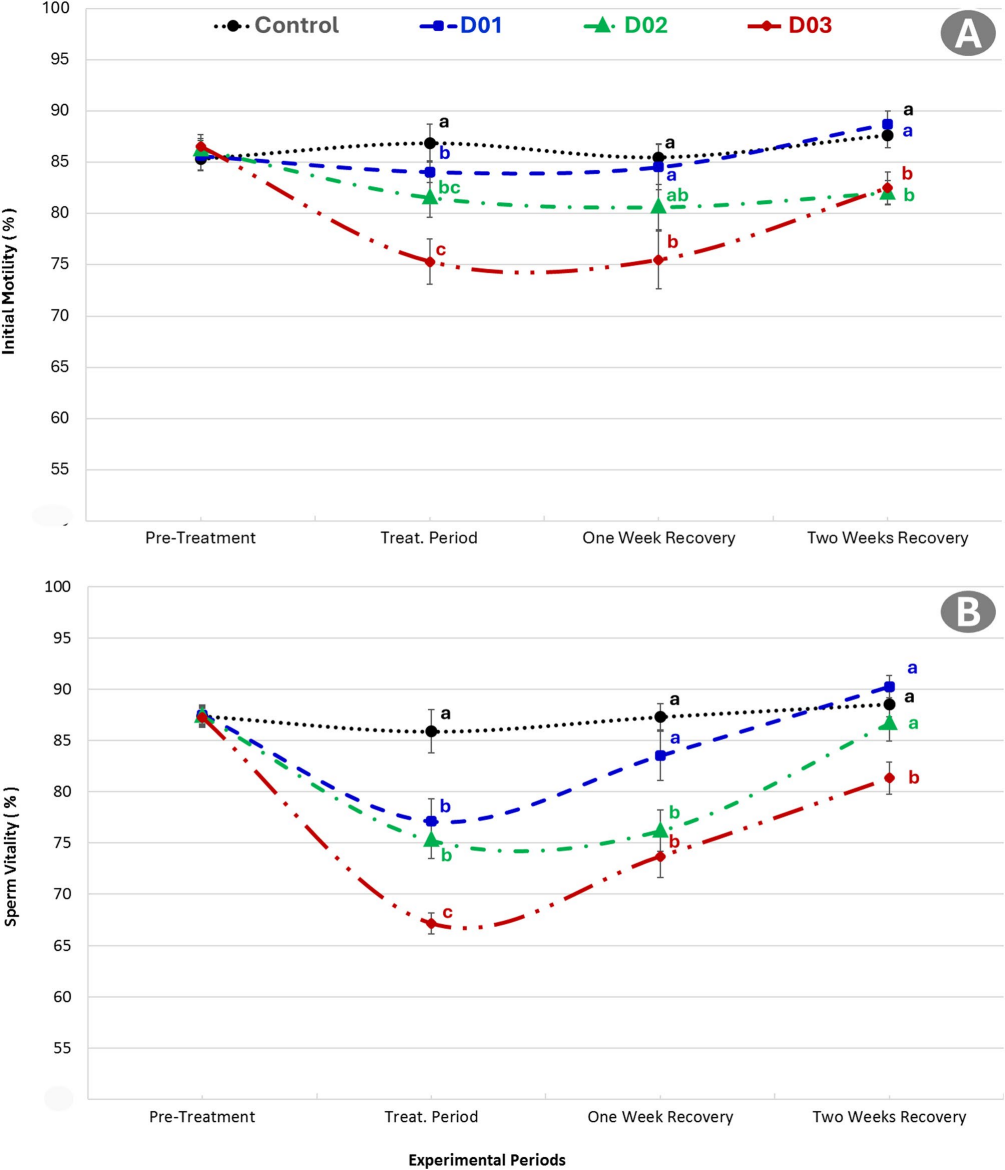
Effect of intramuscular dexamethasone administration at different doses (control, 1, 2 and 3 mg/kg) on initial sperm motility (**A**) and sperm vitality (**B**) in New Zealand White male rabbits. Different superscript letters (a, b, c) indicate statistically significant differences (*p* ≤ 0.05) between groups

Figure 1 showed the effect of different doses of dexamethasone (1, 2 and 3 mg/kg) on reaction time as an indicator of sexual libido and plasma testosterone level. Dexamethasone treatment resulted in a significant (*p* ≤ 0.05) increase in reaction time, indicating a reduction in sexual libido. The effect was dose-dependent, with the highest dose group showing the longest reaction time. Partial recovery occurred after cessation of treatment, with the lower dose group (D01, 1 mg/kg) returning to near baseline levels faster than the higher-dose (D03: 3 mg/kg) group. It continued to exhibit delayed recovery, indicating a more sustained inhibitory effect at higher doses. Blood testosterone concentrations declined significantly in all dexamethasone-treated groups during the treatment period compared to the control group (*P* ≤ 0.05) as presented in Fig. 2. The suppression was dose-dependent, with the D03 group (3 mg/kg) exhibiting the lowest testosterone levels, followed by D02 and D01. During the recovery period, testosterone levels showed partial restoration but remained significantly lower in the D03 group compared to both the control and lower-dose groups (*P* ≤ 0.05). No significant differences were observed between the control and the D01 group by the end of recovery.

### Effect of DEX on semen characteristics and sperm output

Figure 2 clarified the effect of intramuscular dexamethasone administration at different doses (control, 1, 2 and 3 mg/kg) on initial sperm motility and sperm vitality in NZW male rabbits. Results showed a significant (*P* < 0.05) decline in sperm motility, viability, and total sperm output were observed in dexamethasone-treated rabbits, with the most severe impairment in the highest dose group D03 exhibiting the lowest motility (~ 75%). One-week post-treatment, partial recovery was observed, with the D01 group returning closer to control levels. Although recovery was evident over two weeks, the D02 (2 mg/kg) and D03 groups still exhibited significant (*P* < 0.05) improvements but remained lower than control values.

**Fig. 3:**
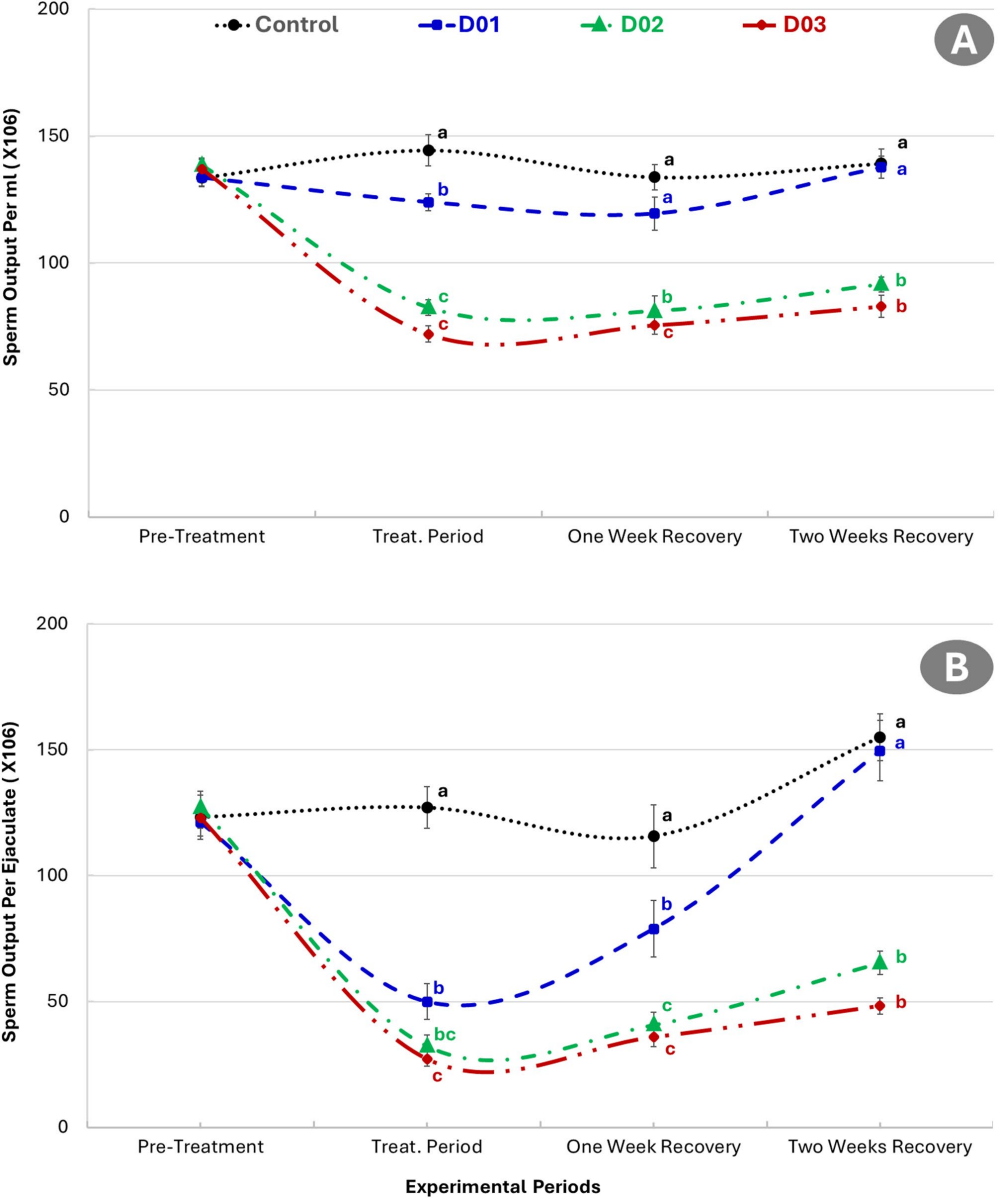
Effects of short-term dexamethasone administration on sperm output (×10^6^) per milliliter (**A**) and per ejaculate (**B**) in New Zealand White male rabbits. Rabbits were treated with dexamethasone intramuscularly at doses of 1 mg (D01), 2 mg (D02), and 3 mg (D03) for one week. Control animals received no treatment. Different letters indicate significant differences between groups within each period (*P* ≤ 0.05)

Figure 3 illustrates the effects of dexamethasone administration on sperm output per milliliter and per ejaculate in NZW male rabbits over different experimental periods. During the pre-treatment phase, no significant differences were observed among all groups. However, during the treatment period, all dexamethasone-treated groups (D01, D02, and D03) showed a significant reduction in sperm output per milliliter and per ejaculate compared to the control group (*P* ≤ 0.05). The most profound reductions were observed in the D02 and D03 groups. Although a gradual improvement in sperm output was seen during the one- and two-week recovery periods, the values in D02 and D03 remained significantly lower than the control, while D01 approached baseline levels. Control animals maintained relatively stable output throughout all periods.

### Effect of DEX on physiological parameters

**Fig. 4:**
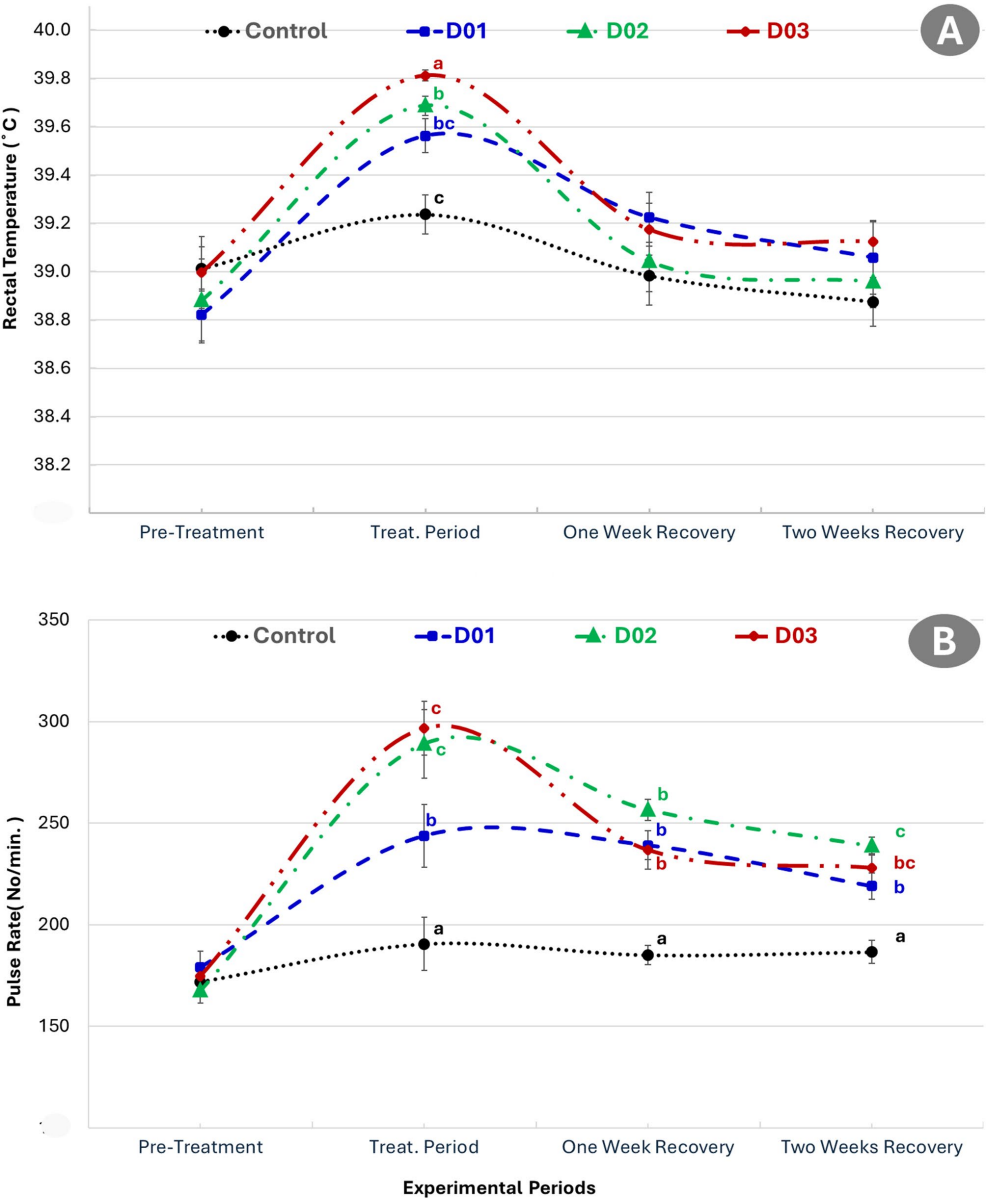
Rectal temperature (**A**) and pulse rate (**B**) in NZW male rabbits across experimental periods after dexamethasone treatment (Control: saline, D01: 1 mg/kg, D02: 2 mg/kg, D03: 3 mg/kg). Different letters indicate significant differences (*P* ≤ 0.05) within the same period

Figure 4 presented the rectal temperature and pulse rate in NZW male rabbits across experimental periods after dexamethasone treatment. Rectal temperature significantly increased during the treatment period in all dexamethasone-treated groups (D01, D02, and D03) compared to the control group (*P* ≤ 0.05). The D03 group exhibited the highest rectal temperature, followed by D02 and D01, indicating a dose-dependent response. Although rectal temperatures declined during the recovery periods, they remained slightly elevated compared to the control group, particularly in D03. Similarly, as presented in the same figure, a marked increase in pulse rate among the dexamethasone-treated groups during the treatment period. The highest pulse rate was observed in the D03 group, followed by D02 and D01, all significantly higher than the control (*P* ≤ 0.05). Pulse rate decreased gradually during the recovery periods but remained significantly higher in the D02 and D03 groups compared to the control at two weeks post-treatment.

### Effect of DEX on hematological parameters

**Fig. 5:**
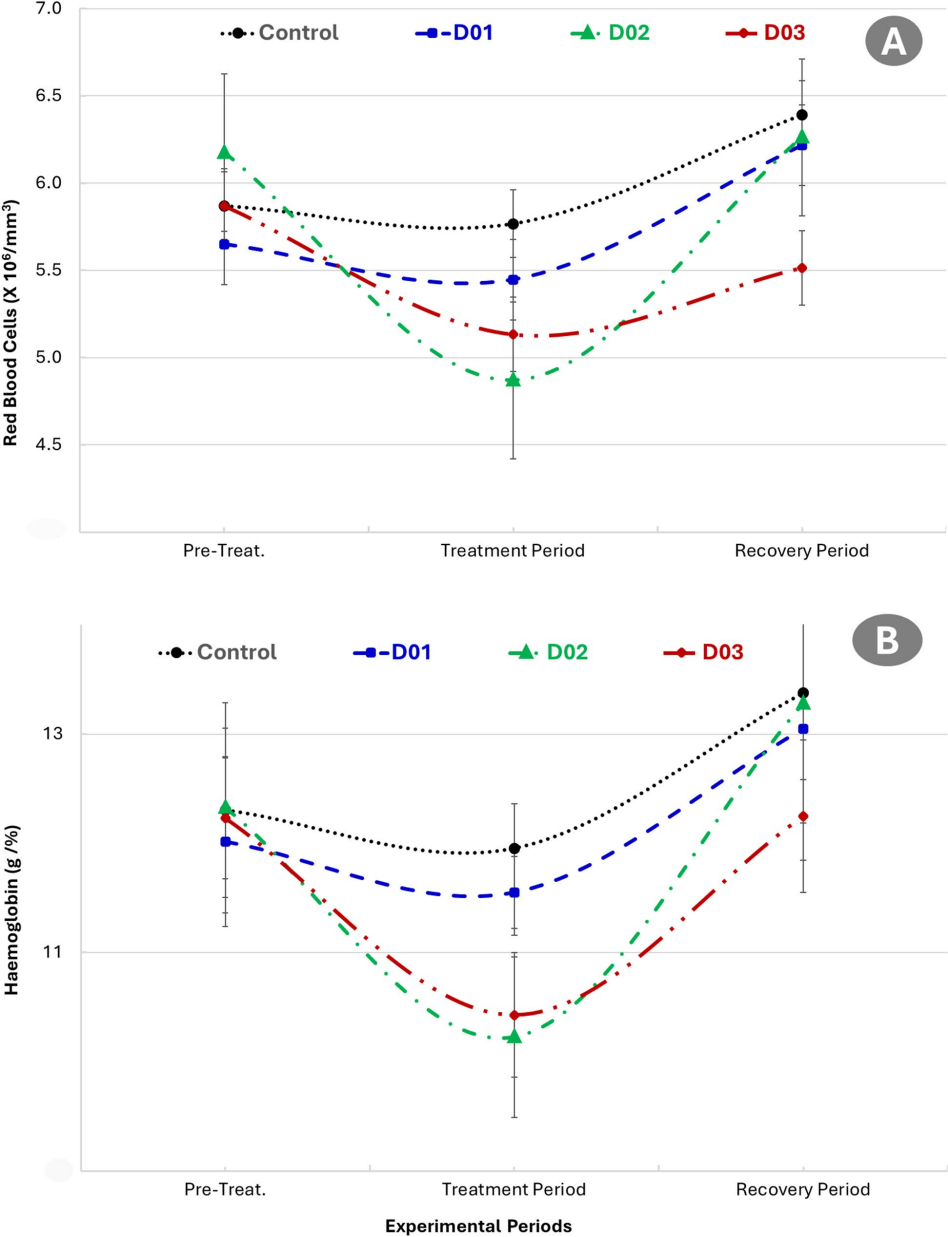
Effect of dexamethasone administration on Red Blood Cells Count (**A**) and haemoglobin concentration (**B**) in male New Zealand White rabbits. Rabbits were treated intramuscularly with dexamethasone at doses of 0 mg (control), 1 mg/kg (D01), 2 mg/kg (D02), and 3 mg/kg (D03) for one week

**Fig. 6:**
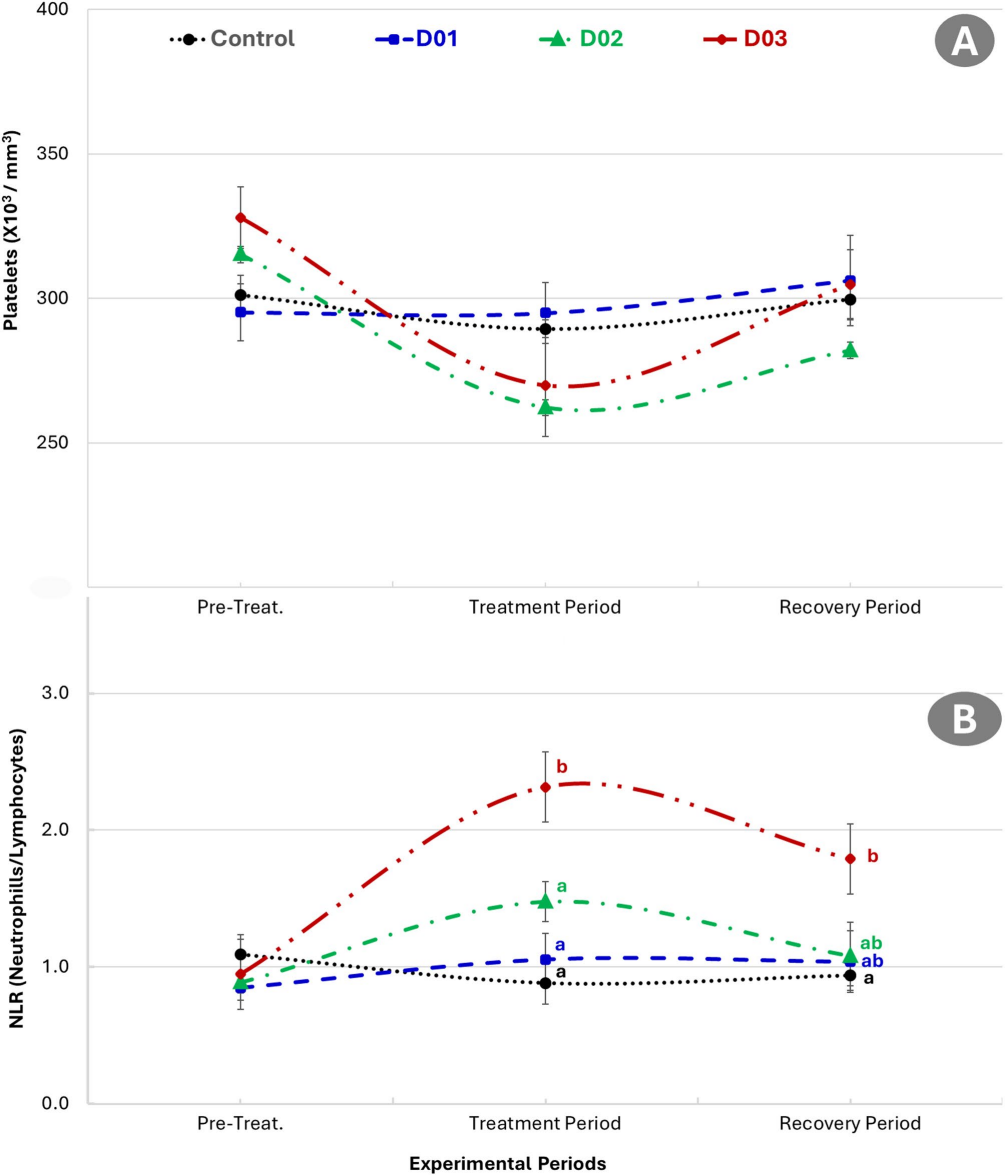
Effect of different dexamethasone doses (0, 1, 2, and 3 mg/kg) on Platelet count (**A**) and NLR, neutrophil-to-lymphocyte ratio, (**B**) in Male New Zealand White Rabbits. Different letters indicate significant differences (*P* ≤ 0.05)

Figure 5 showed the effect of dexamethasone administration on red blood cells (RBCs) count and haemoglobin concentration in male NZW rabbits. A reduction in RBCs count was observed during the treatment period across all dexamethasone-treated groups (D01, D02, and D03) compared to the pre-treatment period. This was followed by an apparent increase in RBCs count during the recovery period. However, no statistically significant differences were detected between the treated groups and the control, or among the treatment groups themselves during any of the experimental periods. Hemoglobin concentrations (Fig. 5) declined during the treatment period in all dexamethasone-treated groups relative to the pre-treatment period and subsequently increased during the recovery phase. Despite these observable trends, there were no statistically significant differences between the treated groups and the control or between different treatment doses. Platelet counts (Fig. 6) decreased slightly in all dexamethasone-treated groups during the treatment period and showed a subsequent increase during recovery. Nevertheless, these variations were not statistically significant.

Figure 6 explained the effect of different dexamethasone doses (0, 1, 2, and 3 mg/kg) on platelet count and neutrophil-to-lymphocyte ratio (NLR) in male NZW Rabbits. The NLR exhibited marked alterations in response to dexamethasone administration across different experimental periods. During the pre-treatment period, no significant differences in NLR values were observed among all groups (control, D01, D02, and D03), indicating similar baseline levels. However, during the treatment period, a dose-dependent elevation in NLR was observed. The D03 group (3 mg/kg) recorded the highest NLR value, significantly greater than both the control and other treatment groups. The D02 group (2 mg/kg) showed a moderate but statistically significant increase in NLR compared to D01 and control, while the D01 group (1 mg/kg) showed no significant difference from the control group. In the recovery period, NLR values decreased in all treated groups but remained elevated in the D03 group, which continued to differ significantly from the control group. The D01 and D02 groups exhibited intermediate NLR values that did not significantly differ from the control or each other. These findings indicate that higher dexamethasone doses lead to greater elevations in NLR, with partial reversibility observed after treatment cessation.

### Effect of DEX on thyroid T3 activity

**Fig. 7:**
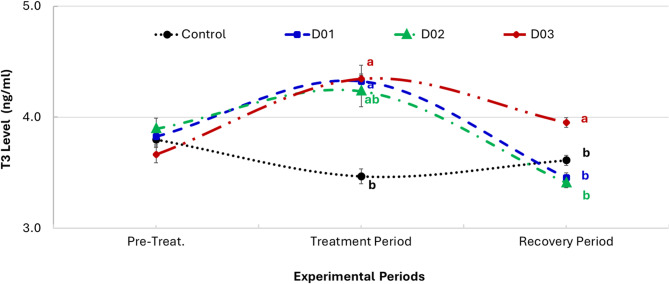
Changes in blood triiodothyronine (T3) concentrations (ng/ml) in New Zealand White male rabbits administered intramuscular dexamethasone at 0 mg (control), 1 mg (D01), 2 mg (D02), and 3 mg (D03) doses per one kg across experimental periods. Different superscript letters denote significant differences between groups (*P* ≤ 0.05)

Figure 7 demonstrates the effects of intramuscular dexamethasone at doses of 0 mg/kg (control), 1 mg/kg (D01), 2 mg/kg (D02), and 3 mg/kg (D03) on blood triiodothyronine (T3) levels in NZW male rabbits during the experimental periods. During the pre-treatment phase, no significant differences in T3 levels were observed between groups, confirming comparable baseline thyroid function. In the treatment period, T3 levels significantly increased in all dexamethasone-treated groups. The D03 group recorded the highest T3 levels, significantly exceeding those of the control and lower-dose groups (*P* ≤ 0.05). The D02 and D01 groups also showed increased T3 concentrations, but the differences were not significant relative to each other or to the control. In the recovery period, a significant decrease in T3 levels was observed in the D01 and D02 groups compared to the control, while the D03 group maintained a higher T3 concentration similar to that of the control, indicating a sustained hormonal response.

### Effect of DEX on rabbit behavior

Rabbits in the open field test from the treated groups reported a significant (*P* ≤ 0.05) increase in anxiety-related responses compared to the control one, as shown in Table 1. High doses of DEX administered in treated group D03 recorded the longest time (s) till rabbits entered the arena, standstill, and grooming while the same group had the lowest exploration time (s) compared to other treated groups and control. Also, there was a highly significant (*P* ≤ 0.01) decrease in time (S) of movement, running, and the frequency of total and central displacements in treated groups when compared to the control. This was obviously detected by the decreased time (S) of latency to enter the arena, standing still, and grooming. This was accompanied by an increase in time (S) of exploration, movement, running, and the frequency of total and central displacements in the control group.

**Table 1:** Open field test results among treated groups at the end of the treatment period

**Treatment Period/Groups**	**Control**	**D01**	**D02**	**D03**	**P-value**
Latency (S) to enter the arena	20.3^a^±0.44	22.5^b^±0.09	22.4^b^±0.26	23.6^c^±0.20	0.02*
Total Displacements	52.1^c^±0.15	49.4^b^±0.25	49.2^b^±0.18	48.0^a^±0.09	0.00**
Central Displacements	4.0^c^±0.02	3.7^b^±0.04	3.6^b^±0.07	3.4^a^±0.01	0.01**
Exploration (S)	479.6^c^±2.60	474.0^b^±0.58	472.0^b^±0.58	468.2^a^±0.58	0.05*
Movement (S)	39.2^c^±0.06	39.1^c^±0.09	38.4^b^±0.09	38.0^a^±0.06	0.00**
Running (S)	7.3^c^±0.03	5.7^b^±0.07	5.7^b^±0.05	5.4^a^±0.02	0.00**
Standing Still (S)	47.3^a^±0.88	51.9^b^±0.06	54.1^c^±0.36	56.6^d^±0.26	0.00**
Grooming (S)	6.3^a^±0.06	6.8^b^±0.09	7.4^c^±0.04	8.2^d^±0.08	0.00**

* Means with different superscripts within the same row are significantly different at the P ≤ 0.05 level

** Means with different superscripts within the same row are significantly different at the P ≤ 0.01 level

**Fig. 8:**
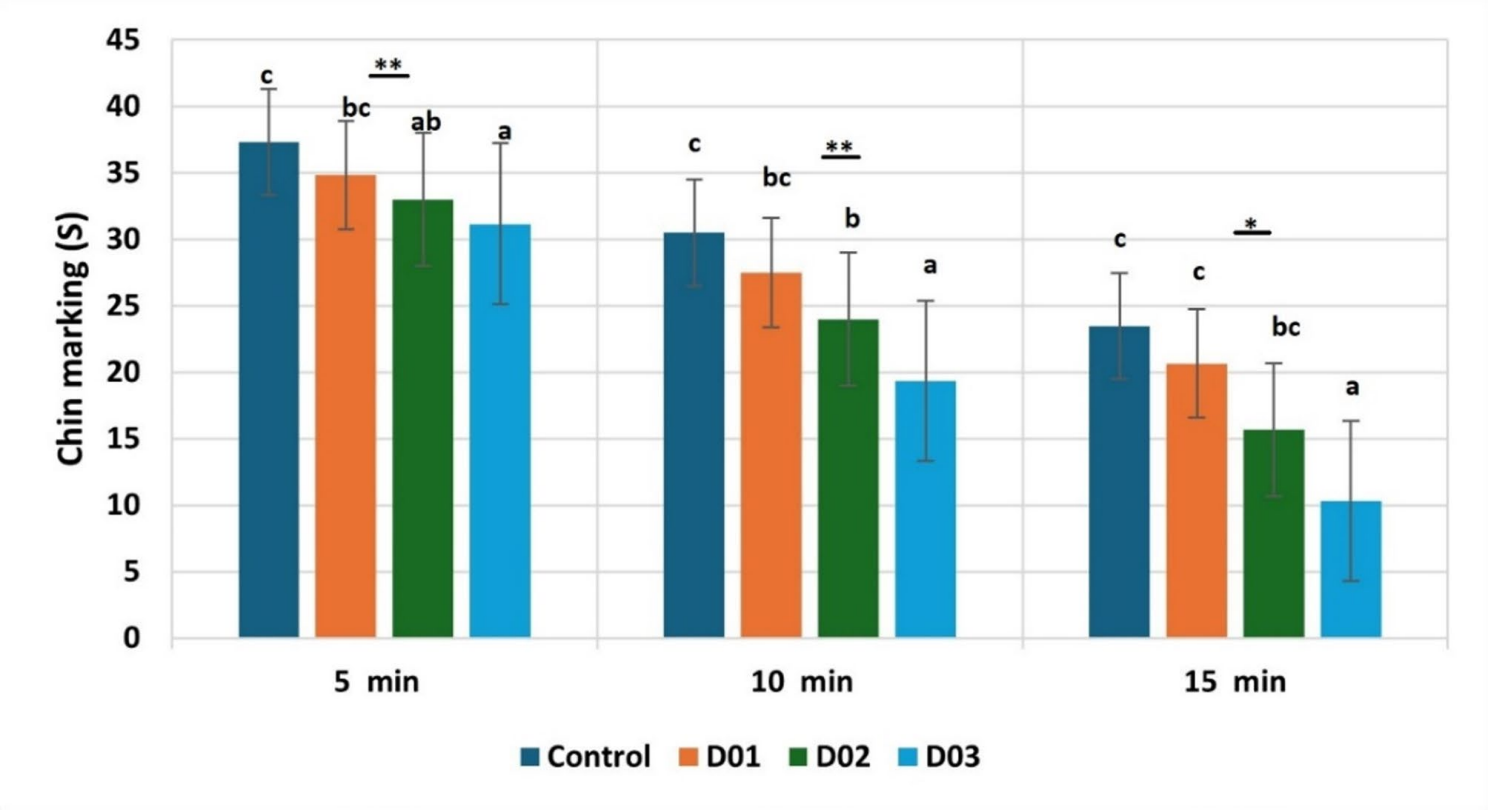
Chin marking frequencies during habituation of NOR test among treated groups across each of the three 5-min of the sample phase. * Means with different superscripts within the same row are significantly different at the *P* ≤ 0.05 level. ** Means with different superscripts within the same row are significantly different at the *P* ≤ 0.01 level

Figure 8 elucidated the chin marking frequencies during habituation of NOR test among treated groups across each of the three 5-min of the sample phase. Results showed that there was a highly significant (*P* ≤ 0.01) and a significant (*P* ≤ 0.05) difference in rabbit chin marking frequencies during habituation of NOR test among treated groups during the first, second, and third 5-min of the sample phase, respectively. Where, the treated group D03 recorded the lowest frequency during the third 5 min of the sample phase when compared to other groups treated and control. There was a non-significant (*P* > 0.05) difference between treated groups D01 and D02 during the three 5-min of the sample phase.

**Fig. 9:**
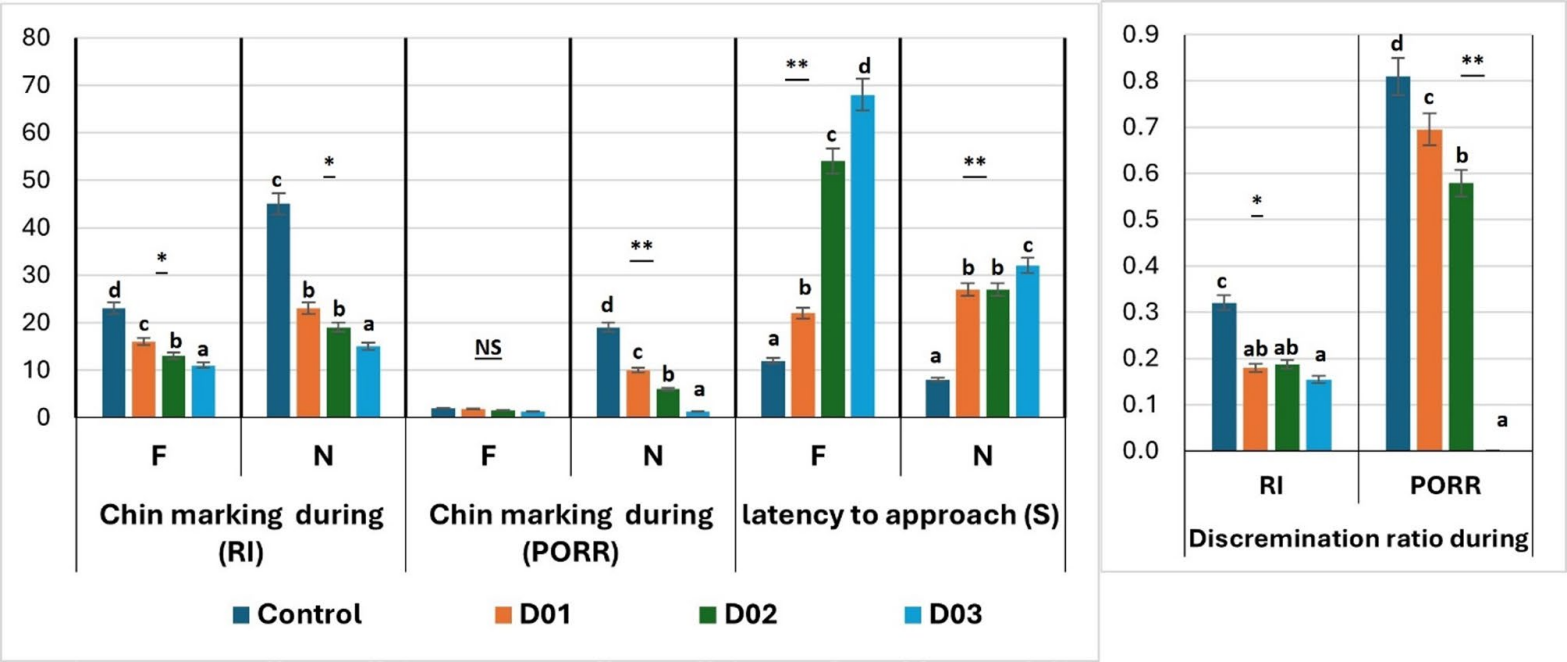
Effect of dexamethasone treatment on object recognition memory during testing phase of NOR. * Means with different superscripts within the same row are significantly different at the *P* ≤ 0.05 level. ** Means with different superscripts within the same row are significantly different at the *P* ≤ 0.01 level. Familiar object (F), Novel object (N), Recognition Interval (RI), Primary Object Recognition Response (PORR)

Figure 9 showed the effect of dexamethasone treatment on object recognition memory during testing phase of NOR. During RI of the testing phase of NOR test after the 5-min delay there was a significant (*P* ≤ 0.05) decrease in rabbit chin marking for both familiar and novel objects among treated groups. The lowest frequencies were recorded in treated group D03 while the highest frequencies were recorded in the control group. Besides, during PORR there was a highly significant (*P* ≤ 0.01) decrease in rabbit chin marking for only the novel objects among treated groups. The lowest frequencies were recorded in treated group D03 while the highest frequencies were recorded in the control group. In contrast, there was a non-significant (*P* > 0.05) difference between treated groups for familiar chin marking.

Results in Fig. 9 showed that there was a highly significant (*P* ≤ 0.01) increase in rabbit latency (S) to approach both familiar and novel objects among treated groups. The lowest duration was recorded in the control group when compared to other treated groups. In addition, there was a highly significant (*P* ≤ 0.01) decrease in rabbit discrimination ratio for both RI and PORR among treated groups when compared to the control group. The lowest rabbit discrimination ratio was recorded in treated group D03 while the highest was recorded in the control group.

### Effect of DEX on body weight changes

**Fig. 10:**
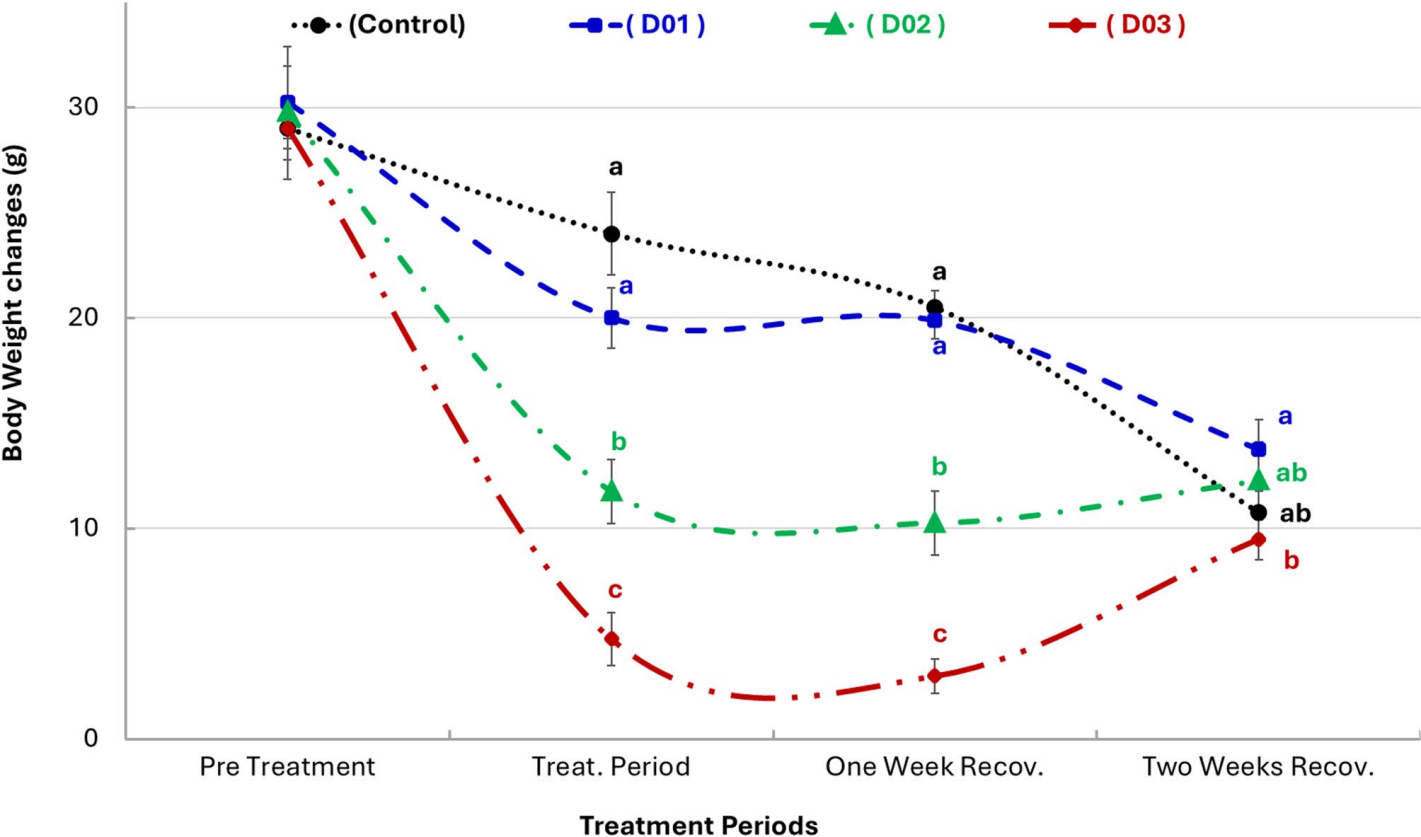
Body weight changes (mean ± SEM) in male New Zealand White rabbits following short-term intramuscular administration of different doses of dexamethasone. Data illustrate the dose-dependent effects of dexamethasone on body weight gain and the partial recovery observed post-treatment. Different letters (a, b, c) denote statistically significant differences (*P* ≤ 0.05) between groups at the same time point

Figure 10 showed body weight changes in male NZW rabbits following short-term intramuscular administration of different doses of dexamethasone. Rabbits treated with dexamethasone showed a significant reduction (*P* ≤ 0.05) in weight gain compared to controls, with the most pronounced effect in the highest dose group (D03, 3 mg/kg). Although recovery occurred over time, the D03 group remained significantly behind the control group in terms of body weight gain by the end of the two-week recovery period. By the end of the second recovery period (two weeks post-treatment), the D01 and D02 groups approached the levels of the control group, whereas the D03 group continued to exhibit relatively lower gains despite some improvement compared to the treatment period.

## Discussion

The administration of DEX, a synthetic glucocorticoid, produced significant dose-dependent alterations in the physiological, reproductive, hematological, and endocrine parameters of growing male NZW rabbits. These changes support the hypothesis that systemic exposure to glucocorticoids, especially during sensitive developmental stages, can compromise growth, immunity, and fertility.

The observed alterations in sexual response times are attributed to the well-documented physiological effects of dexamethasone, a potent glucocorticoid receptor agonist. Dexamethasone suppresses the HPG axis by inhibiting the secretion of gonadotropin-releasing hormone from the hypothalamus, leading to decreased levels of LH and follicle-stimulating hormone (FSH), and consequently, reduced testosterone production [47, 48]. In addition to central suppression, dexamethasone also directly impairs Leydig cell function within the testes, further diminishing testosterone biosynthesis [49]. These combined effects on the central and peripheral regulatory systems account for the dose-dependent increases in reaction time observed during treatment. The differences in recovery dynamics among groups reflect the extent of endocrine suppression: animals receiving lower doses (D01 and D02) demonstrated a faster rebound in sexual activity, while those exposed to the highest dose (D03) experienced prolonged suppression, consistent with the known cumulative and dose-related inhibitory effects of glucocorticoids [50].

A marked decline in blood testosterone during and post-treatment was observed, especially at 2 and 3 mg/kg doses. This suppression aligns with studies showing DEX-mediated inhibition of LH release and direct Leydig cell suppression via glucocorticoid receptor signaling [51, 52]. Although a partial recovery of testosterone levels was observed after cessation of dexamethasone, the incomplete normalization, particularly in the D03 group, suggests lasting alterations or a delayed recovery of the HPG axis function, as similarly reported in long-term glucocorticoid exposure studies [53, 54]. These findings highlight the sensitivity of reproductive hormonal balance to glucocorticoid administration and underscore the importance of dose consideration when dexamethasone is used therapeutically. These hormonal alterations likely contribute to reduced sexual behavior and prolonged reaction times, as testosterone is crucial for maintaining libido and male sexual performance in rabbits and other mammals [55, 56]. The partial rebound observed in the D01 and D02 groups indicates potential for recovery, although full restoration may require more time or supportive hormonal therapy.

Semen parameters including sperm motility, vitality and output were markedly impaired following DEX treatment. These findings confirm the suppressive effects of glucocorticoids on testicular function through HPG axis disruption and direct toxicity to leydig and sertoli cells [31, 32]. DEX reduces LH and FSH secretion, which leads to lowered testosterone synthesis and impaired spermatogenesis [26]. Notably, sperm motility and concentration continued to be significantly suppressed even after two weeks of recovery, indicating prolonged or possibly irreversible damage at higher DEX doses. These observations are consistent with earlier studies in rams and dogs showing reduced sperm output, increased abnormal forms, and suppressed seminal plasma enzyme activities [57, 58].

DEX induced a transient rise in rectal temperature and pulse rate during the treatment period, followed by normalization in recovery phases. These elevations may reflect increased thyroid hormone activity and metabolic stimulation, as DEX has been shown to upregulate serum T3 levels [59]. Increased cardiovascular activity and pulse rate are known consequences of thyroid hormone action on cardiac myocytes and vasculature [60], although excessive stimulation could predispose to cardiovascular risks.

A non-significant reduction in RBCs count, hemoglobin, and platelet count was observed during the treatment period, particularly in the higher-dose groups, with partial recovery later. These findings are consistent with glucocorticoid-induced myelosuppression and possible erythropoiesis inhibition via suppression of erythropoietin and bone marrow activity [20]. The decline in RBCs following the treatment provides additional evidence and substantiates a significant correlation with testosterone concentrations. Testosterone stimulates erythropoietin (EPO) production in the kidneys, promoting erythroid progenitor cell proliferation and maturation in the bone marrow, thus increasing RBC count or packed cell volume (PCV). It may also suppress hepcidin, enhancing iron availability for erythropoiesis [61–63]. Although the changes were not statistically significant, they suggest subclinical hematological compromise. The partial restoration of hematological parameters during the recovery period implies that these changes were reversible following cessation of dexamethasone treatment. However, slight decreases in the higher dose groups may indicate a longer recovery time is needed after more intensive glucocorticoid exposure. Overall, while the findings did not reach statistical significance, the observed patterns are consistent with the literature on the hematological impact of glucocorticoid administration and highlight the importance of monitoring blood parameters even in short-term therapeutic protocols. NLR increased significantly in the 2 and 3 mg/kg DEX groups during the treatment phase, reflecting an immunosuppressive shift in leukocyte profiles. The elevation is indicative of neutrophilia and lymphopenia, hallmark effects of glucocorticoid action on circulating immune cells [11]. This shift also points to increased systemic stress and inflammation-like responses under corticosteroid treatment. Furthermore, NLR elevation during the treatment phase suggests a strong immunomodulatory and anti-lymphocytic effect of dexamethasone, consistent with its known glucocorticoid actions, including neutrophil demargination and lymphocyte apoptosis [64].

Although T3 levels rose transiently during treatment, potentially due to HPT axis stimulation, they declined sharply afterward, likely due to feedback inhibition or thyroid tissue damage [65, 66]. These findings reinforce the complex role of glucocorticoids in modulating thyroid hormone metabolism and highlight the importance of dose consideration in therapeutic or experimental settings involving corticosteroids.

Rabbit behaviours in the open field test showed nearly similar data and there were no differences between the control and the three treated groups at the end of the preliminary period. But, after dexamethasone was intramuscularly injected into treated rabbit groups there were great differences among tested groups in rabbit latency to enter the arena (S), and exploration behavior (S). The treated group D03 recorded the longest time till rabbits entered the arena while the same group had the lowest exploration time. In contrast, the control group recorded the shortest time till rabbits entered the arena and it had the highest exploration time. Hand by hand, there were large differences among tested groups of rabbits in the rest of the tested behaviors; total displacements, central displacements, movement (S), running (S), standing still (S), and grooming (S). These clear differences indicated that DEX treated rabbit groups have a pronounced stress response in OFT. These findings agreed with [11]. On the other hand, these findings disagreed with [8] who noted that no behavioral changes were noted for either the DEX-treated or the control groups.

Concerning rabbit chin marking frequencies during the habituation of NOR test, there was a gradual decrease among treated groups from the first 5-min until the third one. Treated group D03 recorded the lowest frequency during the three 5-min of the sample phase when compared to other groups treated and controlled. This indicates that DEX induced a stressful situation in treated groups of high doses leading to the lowered chin marking behavior. These results agreed with Hoffman et al. [67], which showed that this behavior habituated across the short-term (0–15 min). Also, Hoffman and Basurto [22] confirmed these results as habituation response occurred during the sample phase.

In addition, during RI of the testing phase of NOR test after the 5-min delay rabbit chin marking gradually decreased for both familiar and novel objects among treated groups as the highest frequencies were recorded in the control group. So that, there was a stressful response in DEX treated groups and this agreed with Calefi et al. [15] who reported that dietary DEX can produce homologous effects like increased corticosterone levels activating stress-related signaling pathways. Besides, another decrease was recorded during PORR in rabbit chin marking for only the novel objects among treated groups. In contrast, there was no difference between treated groups for familiar chin marking. This finding indicates that memory is used when object recognition is spontaneous and based only on object novelty [68, 69].

Concerning rabbit latency (S) to approach both familiar and novel objects during NOR test there was a slower approach among treated groups than that recorded in the control group. In addition, there was an obvious decrease in rabbit discrimination ratio for both RI and PORR among treated groups when compared to the control group. The worst rabbit discrimination ratio was recorded in treated group D03 while the best was recorded in the control group. This finding indicated that stress could impair memory in rabbits and lower the object discrimination. In agreement with some studies [70, 71] who reported that chronic stress from repeated or prolonged exposure to stressors, tends to have more detrimental effects on cognition and impair spatial memory [72].

DEX-treated rabbits showed a significant reduction in weight gain, especially at higher doses (2 and 3 mg/kg), during the treatment period. This catabolic response aligns with earlier reports highlighting DEX’s ability to enhance protein breakdown, suppress appetite, and reduce energy storage [73, 74]. Despite some studies reporting increases in body weight [59], the reduction observed here is consistent with glucocorticoid-induced growth retardation via hypothalamic-pituitary-somatotropic axis suppression [75]. Moreover, dexamethasone markedly enhances protein catabolism in skeletal muscle by upregulating proteolytic pathways and concurrently suppresses protein synthesis, leading to a net loss of muscle mass or a significant attenuation of muscle accretion. These mechanisms contribute to the reduced rates of body weight gain observed during the treatment period. In addition, dexamethasone has been shown to inhibit the secretion of key anabolic hormones, notably growth hormone and insulin-like growth factors, thereby further suppressing anabolic processes essential for normal growth and development [76, 77]. The differential recovery responses observed among the treatment groups can be explained by the gradual reestablishment of glucocorticoid homeostasis following cessation of dexamethasone administration. Animals treated with lower doses (1 and 2 mg/kg) exhibited a faster recovery of anabolic activities, potentially due to a lesser degree of HPA axis suppression, compared to animals exposed to the highest dose (3 mg/kg), where prolonged HPA axis disruption likely contributed to the sustained impairment of growth even after two weeks of recovery [64, 78].

## Conclusion

Collectively, the study demonstrates that dexamethasone, even when administered for a short period, can exert profound and largely detrimental effects on growth, reproduction, immunity, and endocrine balance in growing male rabbits. These findings reinforce the need for cautious use of glucocorticoids in breeding animals, especially during developmental windows. Recovery trends suggest some reversibility of effects at lower doses; however, high-dose exposure may lead to long-term impairment. Future studies are warranted to explore the underlying molecular mechanisms of these effects, the long-term reproductive consequences, and possible protective strategies to mitigate glucocorticoid-induced damage.

## Data Availability

The datasets used and analyzed during the current study are available from the corresponding author upon reasonable request.
